# 3D kernel-density stochastic model for more personalized glycaemic control: development and in-silico validation

**DOI:** 10.1186/s12938-019-0720-8

**Published:** 2019-10-22

**Authors:** Vincent Uyttendaele, Jennifer L. Knopp, Shaun Davidson, Thomas Desaive, Balazs Benyo, Geoffrey M. Shaw, J. Geoffrey Chase

**Affiliations:** 1 Department of Mechanical Engineering, University of Canterbury, Private Bag 4800, Christchurch, New Zealand; 2 GIGA-In Silico Medicine, University of Liège, Allée du 6 Août 19, Bât. B5a, 4000 Liège, Belgium; 3 Department of Control Engineering and Information Technology, Budapest University of Technology and Economics, Budapest, Hungary; 4 Christchurch Hospital, Dept of Intensive Care Christchurch, New Zealand and University of Otago, School of Medicine, Christchurch, New Zealand

**Keywords:** Glycaemic control, Hyperglycaemia, Blood glucose, Insulin, Insulin sensitivity, Kernel density

## Abstract

**Background:**

The challenges of glycaemic control in critically ill patients have been debated for 20 years. While glycaemic control shows benefits inter- and intra-patient metabolic variability results in increased hypoglycaemia and glycaemic variability, both increasing morbidity and mortality. Hence, current recommendations for glycaemic control target higher glycaemic ranges, guided by the fear of harm. Lately, studies have proven the ability to provide safe, effective control for lower, normoglycaemic, ranges, using model-based computerised methods. Such methods usually identify patient-specific physiological parameters to personalize titration of insulin and/or nutrition. The Stochastic-Targeted (STAR) glycaemic control framework uses patient-specific insulin sensitivity and a stochastic model of its future variability to directly account for both inter- and intra-patient variability in a risk-based insulin-dosing approach.

**Results:**

In this study, a more personalized and specific 3D version of the stochastic model used in STAR is compared to the current 2D stochastic model, both built using kernel-density estimation methods. Fivefold cross validation on 681 retrospective patient glycaemic control episodes, totalling over 65,000 h of control, is used to determine whether the 3D model better captures metabolic variability, and the potential gain in glycaemic outcome is assessed using validated virtual trials. Results show that the 3D stochastic model has similar forward predictive power, but provides significantly tighter, more patient-specific, prediction ranges, showing the 2D model over-conservative > 70% of the time. Virtual trial results show that overall glycaemic safety and performance are similar, but the 3D stochastic model reduced median blood glucose levels (6.3 [5.7, 7.0] vs. 6.2 [5.6, 6.9]) with a higher 61% vs. 56% of blood glucose within the 4.4–6.5 mmol/L range.

**Conclusions:**

This improved performance is achieved with higher insulin rates and higher carbohydrate intake, but no loss in safety from hypoglycaemia. Thus, the 3D stochastic model developed better characterises patient-specific future insulin sensitivity dynamics, resulting in improved simulated glycaemic outcomes and a greater level of personalization in control. The results justify inclusion into ongoing clinical use of STAR.

## Background

Stress hyperglycaemia, or abnormal elevated blood glucose (BG) concentrations resulting from metabolic stress response to injury, is a common complication in critically ill patients, associated with increased morbidity and mortality [1–4]. Glycaemic control (GC) using insulin therapy to reduce BG to safer concentrations has shown improved outcomes, reducing organ failure, clinical burden, and costs [5–10]. However, other studies failed to replicate these results [11–16], showing increased glycaemic variability and higher risk of hypoglycaemia, independently associated with severe complications and death [17–21]. GC has been hard to achieve safely and effectively, often lacking patient specificity and failing to account for inter- and intra-patient variability [22], showing the critical need for model-based patient-specific GC solutions.

To date, the optimal target band for GC is still being debated [23]. Most intensive care units (ICUs) use a higher target band than the normoglycaemic range as a ‘first do not harm’ approach, hypoglycaemia being more harmful [17] for the patient than the potential benefits from GC. However, these standards are based on studies failing to provide safe, effective control for all patients when targeting a lower glycaemic band [24]. In fact, the association between mortality and glycaemic levels, safety, and variability has been shown a function of the control provided and not patient condition [25].

Hence, GC design is the key factor in patient GC outcomes. Failing to achieve safe, effective control for all, regardless of the target band, could bias study results and conclusions [26, 27]. More recently, new studies showed the possibility to achieve safe GC to lower target bands for reduced workload, without increasing hypoglycaemia [28–30]. These recent analyses show intensive GC to lower target bands is possible in critically ill patients, and emphasise, once again, the importance of safe, effective model-based and patient-specific GC protocol design.

Stochastic-TARgeted (STAR) is a validated model-based GC framework, which has shown effective control in three different countries [28]. STAR uses a physiological model to describe the glucose–insulin dynamics, and a population-based stochastic model to directly account for patient-specific future metabolic variability [31]. Patient-specific insulin sensitivity (SI) key parameter [22, 32], describing patient metabolic response to insulin, is identified from clinical data and its future potential variability assessed to adjust treatment according to potential risks. This unique patient-specific risk-based dosing approach minimizes the risk of hypoglycaemia while providing effective GC [28, 31].

The quality of control resides in the ability to adapt treatment to patient-specific needs, which is a function of the level of difficulty to control [25]. The difficulty to control is mainly captured by SI variability [33], where variability extremes can lead to hyper- or hypoglycaemia for a given insulin intervention. In STAR, SI is considered constant over 1-h periods, and the variability is assessed by the hour-to-hour change in SI levels. The prediction of future SI evolution is thus a key element for the quality of GC, which needs to be well addressed, especially since SI variability is equivalent among patients with different clinical outcomes [25].

STAR predicts future SI evolution using stochastic models [34]. The stochastic model in STAR forecasts future SI (SI_*n*+1_) distributions based on the current identified SI value (SI_*n*_). However, the current stochastic models [34–36] are potentially over-conservative due to large prediction bands. Wide prediction bands can limit insulin dosing, resulting in lower insulin doses to avoid stochastically forecasted hypoglycaemic risk. Better control might be obtained from a more detailed, and thus more personalized stochastic models. This study aims to improve SI variability forecasting using additional data information.

Using prior temporal information of SI evolution has shown better prediction accuracy [37]. Using such model gives generally tighter prediction bands at a given SI level, allowing potentially higher insulin rates for the patient. While encouraging, the method in [37] lacks model resolution and definition, making comparison hard with the current 2D stochastic model used in STAR. This study thus more specifically aims to develop a new 3D stochastic model accounting for prior knowledge of SI evolution, using 2 inputs (SI_*n*_ and SI_*n*−1_) to determine likely future SI_*n*+1_. The added input can provide higher patient specificity, allowing more accurate insulin dosage for the patient. More specifically, a wider future prediction range for SI would suggest higher potential variability; thus, lower insulin rates will likely be recommended. In contrast, tighter prediction bands would suggest lower variability and thus, potentially higher insulin recommendation. In this study, the new 3D stochastic model is generated using multivariate kernel-density estimation method similar to the one used for the current 2D. This will improve model resolution. In addition, this study assesses the impact of this new presented 3D stochastic model on GC performances, using virtual trial simulations.

## Results

### 2D vs. 3D models forward predictive power

**Fig. 1:**
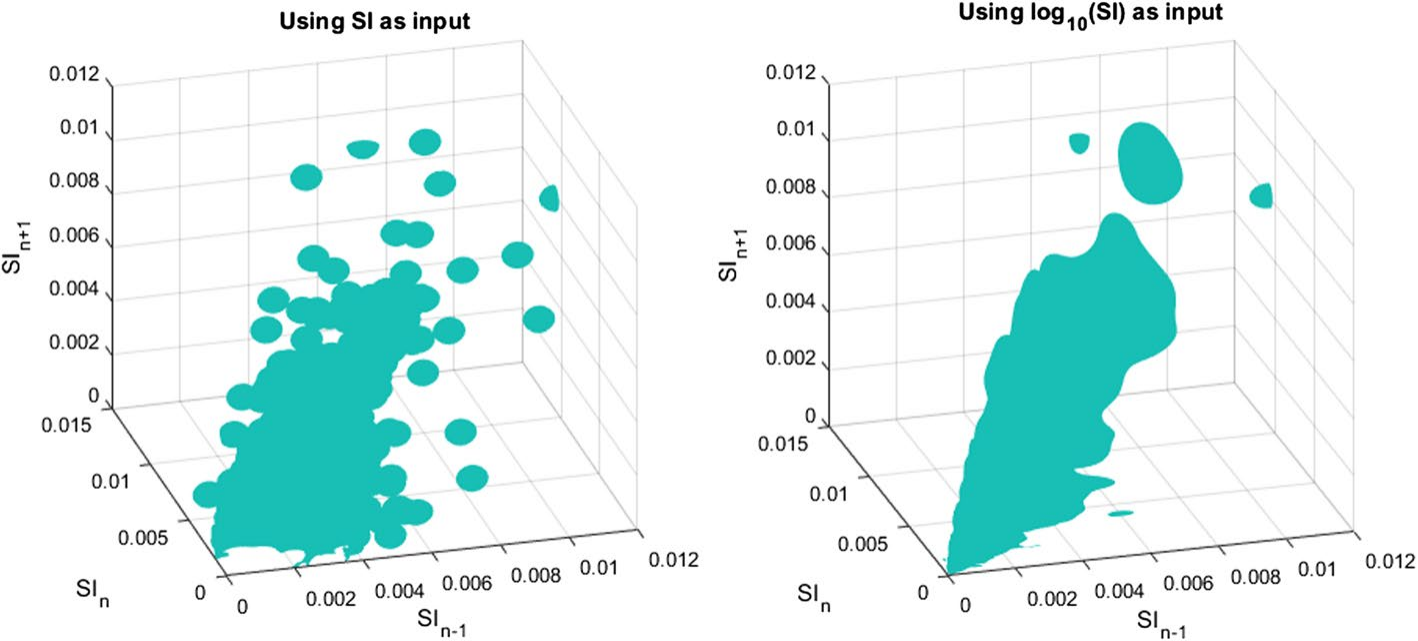
Graphical representation of kernel-density estimation using normal data (left) or logarithmic-transformed data (right)

A representation of the kernel-density estimation is shown in Fig. 1. The left panel shows the kernel-density surface using the normal data, whereas the right panel shows the kernel-density surface when data are transformed into the log-normal space to meet the normal distribution assumption under Silverman’s rule of thumb (ROT) [38]. Clearly, log-normal data provide increased data density for higher SI ranges, where the raw data are sparser. Hence, this approach, taken for the first time here, potentially improves safety by better characterising SI potential variability for higher SI ranges, where the risk of experiencing hypoglycaemia due to insulin dosing is greater.

**Fig. 2:**
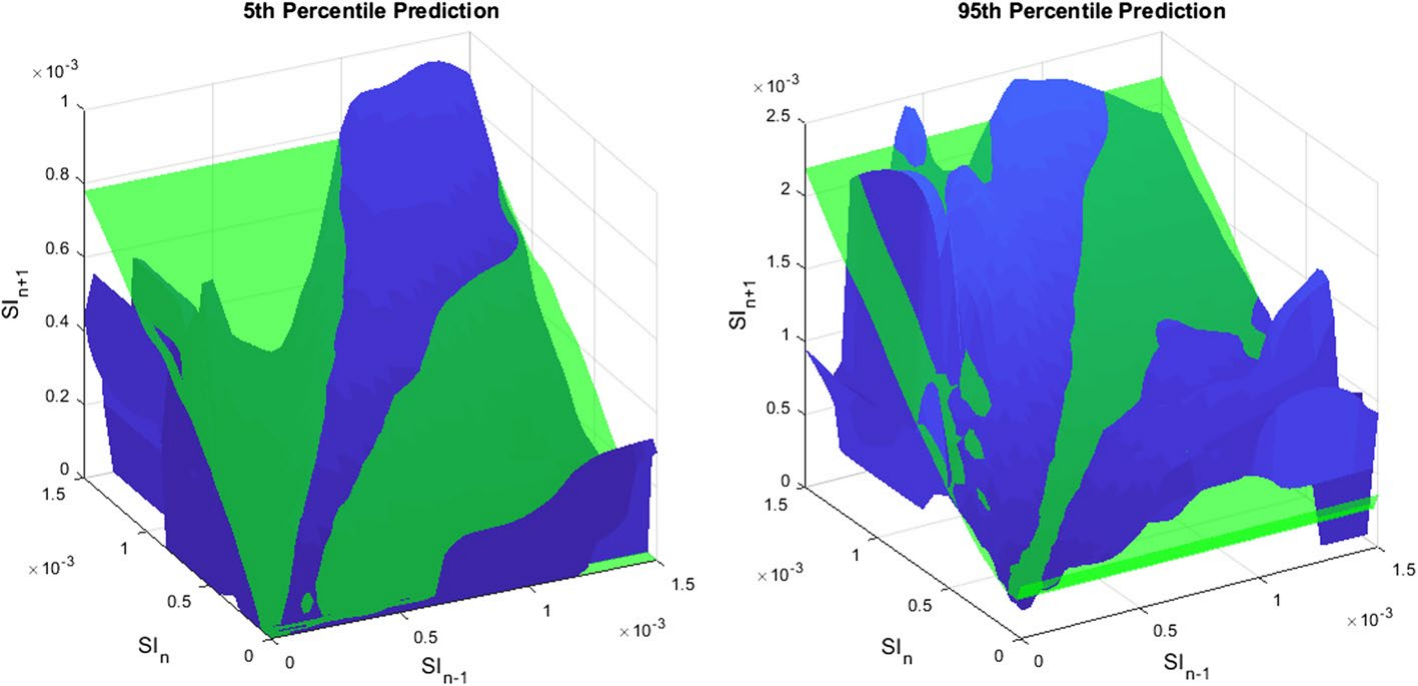
Comparison between 5th (left) and 95th (right) percentile predictions of likely future SI for the 2D model (green) and the 3D model (blue). The 2D model is constant across SI_*n*-1_ whereas the 3D model varies

**Fig. 3:**
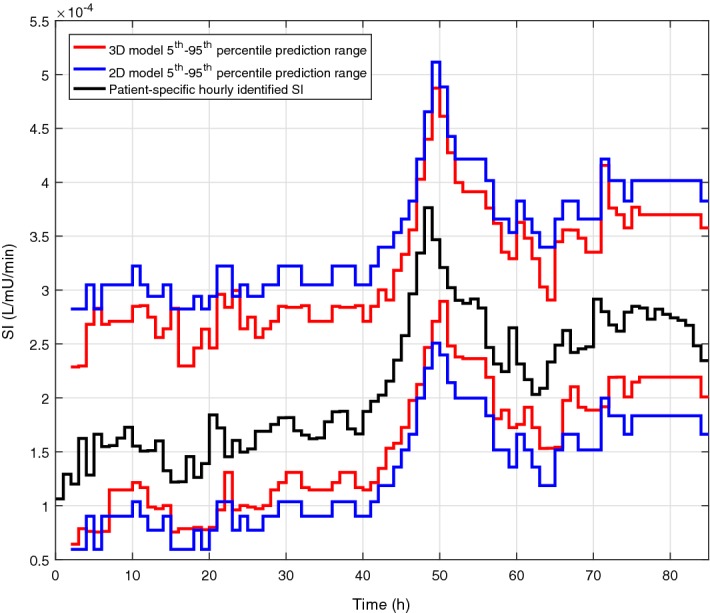
Excerpt of SI evolution (black) and corresponding 2D (blue) and 3D (red) forward prediction ranges for specific virtual patient. The 3D model prediction ranges are generally narrower

**Fig. 4:**
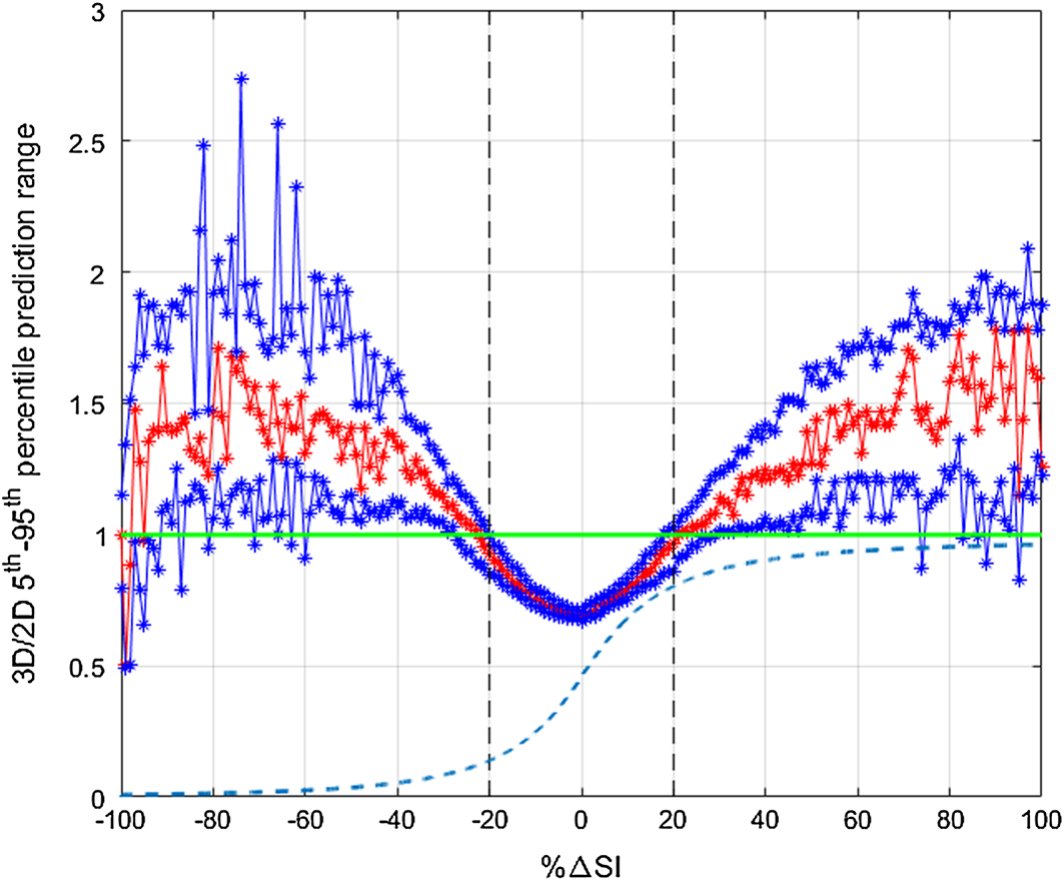
Median [IQR] ratio between the 3D and 2D model 5th–95th percentile prediction width as a function of the hour-to-hour percentage change in SI (%ΔSI). The cumulative distribution function of  %ΔSI is also shown in the blue dashed line

Cross-validation results’ summary of the forward predictive power for both models is presented in Table 1. In addition, the resulting 5th and 95th percentile predictions for each model are shown in Fig. 2. Both the 2D and 3D models have close to 50% (~ 53% vs. ~ 51%) and 90% (~ 91% vs. ~ 90%) predictions in the 25th–75th and 5th–95th percentile ranges, respectively. However, the prediction ranges are generally narrower (~ 70% of hours) in the case of the 3D model. An example of the evolution of SI for a patient and the 2D and 3D predictions ranges for a specific virtual patient is shown in Fig. 3. In addition, the median [IQR] percentage predictions in the 25th–75th and 5th–95th percentile prediction ranges are closer to the expected 50% and 90% for the 3D model, suggesting that the 2D model is too conservative for most patients. To characterise the difference in prediction ranges from both models, the percentage change in the 5th–95th percentile range widths is computed for every prediction and the median [IQR] of percentage change is reported in Table 1. The high prediction performances are achieved with significantly 15.5–24.4% tighter 5th–95th percentile prediction range 69.9–73.8% of the time and 14.8–22% wider otherwise. The median [IQR] 3D/2D prediction width ratios as a function of the hour-to-hour percentage change in of SI (%ΔSI) are shown in Fig. 4, where clearly, prediction bands are typically tighter when  %ΔSI is within ± 20%. Overall, the new model thus better captures patient-specific differences from this more optimal model.

**Table 1:** Fivefold cross-validation results’ summary of forward predictive power and prediction range comparison between 2D and 3D stochastic models

	Total predictions	1-hourly	2-hourly	3-hourly
		58,539	57,840	57,141
2D model	% predictions in 25th–75th	55.9	53.4	52.6
	% predictions in 5th–95th	91.4	91.0	91.0
3D model	% predictions in 25th–75th	52.6	51.3	51.0
	% predictions in 5th–95th	90.5	90.2	90.2
3D vs. 2D model	% of tighter predictions using 3D model	73.8	72.8	69.9
	% reduction in 5th–95th prediction width	24.4 [17.7 29.4]	17.9 [10.9 20.9]	15.5 [10.8 19.2]
	% of wider predictions using 3D model	26.2	27.2	30.1
	% increase in 5th–95th prediction width	22.0 [7.5 49.1]	16.4 [7.7 32.0]	14.8 [6.8 28.2]

Data given as median [IQR] where appropriate

### Virtual trials’ results

Virtual trial results of STAR using the two different stochastic models are summarised in Table 2. Overall, both versions of STAR provided similar performance in terms of median BG [IQR] (6.3 [5.7, 7.0] vs. 6.2 [5.6, 6.9] mmol/L) and percentage time in the 4.4–8.0 mmol/L target band (88%). However, the overall  %BG measurements shifted toward lower BG ranges using STAR-3D, with significantly higher  %BG within 4.4–6.5 mmol/L and 4.4–7.0 mmol/L (61% vs. 56% and 75% vs. 72%, *p* < 0.01 using *χ*^2^ statistical test on proportions of measurements). In terms of safety, both models excel similar to only 2% BG < 4.4 mmol/L, 1% BG < 4.0 mmol/L, and 0.03% BG < 2.2 mmol/L, despite STAR-3D administering higher median insulin (3.0 [1.5, 5.0] vs. 2.5 [1.5, 4.5] U/h). Slightly lower, but similar,  %BG in 8–10 mmol/L (mild hyperglycaemia) for STAR-3D is also observed (7% vs. 8%). Finally, STAR-3D provided higher goal feed (97 [36, 100] vs. 95 [40, 100]  %GF]).

**Table 2:** Virtual trial results summary for STAR-2D and STAR-3D

	STAR-2D	STAR-3D
Number of patients	681	681
Hours of control (h)	59,073	59,071
Total BG measurements	31,248	31,858
Workload (measurements per day)	12.7	12.9
Median [IQR] BG (mmol/L)	6.3 [5.7 7.0]	6.2 [5.6 6.9]
% BG in 4.4–6.5 mmol/L	56	61
% BG in 4.4–7.0 mmol/L	72	75
% BG in 4.4–8.0 mmol/L	88	88
% BG in 8.0–10.0 mmol/L	8	7
% BG > 10.0 mmol/L	3	3
% BG < 4.4 mmol/L	2	2
% BG < 4.0 mmol/L	1	1
% BG < 2.2 mmol/L	0.03	0.03
# patients < 2.2 mmol/L	11 (1.6%)	11 (1.6%)
Median [IQR] insulin rate (U/h)	2.5 [1.5 4.5]	3.0 [1.5 5.0]
Median [IQR] dextrose rate (%GF)	95 [40 100]	97 [36 100]

## Discussion

One of the key factors making GC difficult is patient variability. The risk-based dosing approach used in STAR relies on stochastic forecasting of likely future variation of SI, which is used to assess the risks of hyper- and, more importantly, hypo-glycaemia, and select appropriate treatment based on this risk. If the 5th–95th percentile in SI prediction range is narrowed, control can be improved using more aggressive insulin dosing and/or greater dextrose intake to safely reach the same or lower glycaemic range. On the other hand, if the 5th–95th percentile prediction range for the 3D stochastic model is widened, it suggests greater potential variability in future SI, leading to less aggressive insulin dosing to overcome the higher risk of hypoglycaemia.

The comparison between the 2D and 3D models clearly shows the new model accuracy to predict future SI, with 15.5–24.4% tighter prediction range for more than 69.9–73.8% of the hours (Table 1). Typically, the prediction range is tighter when  %ΔSI is within ± 20% (Fig. 4). On the contrary, the prediction range is wider when the variation is larger than ± 20%. This key outcome thus suggests that the previous patient-specific metabolic variability has a direct impact on future SI forecasting. More specifically, this 3D model shows stable patients, with low previous variation in SI, and tends to remain stable, whereas more variable patients are more likely to have bigger future metabolic variations, as clearly shown in Figs. 2 and 3. Hence, the 2D stochastic model is over-conservative in terms of insulin intervention for most patients. The 3D approach allows STAR to select more aggressive insulin dosing more than 69.9% of the time, while ensuring safety, using the proven risk-based dosing approach. Therefore, the resulting greater patient specificity implies better GC with lower glycaemic variability, and improved glycaemic outcomes.

Virtual trial results comparing STAR using the 2D and the 3D stochastic models confirmed these observations showing higher percentage time in normoglycaemic ranges, with 5% more time spent in the 4.4–6.5 mmol/L range, for similar incidence of mild hypoglycaemia (BG < 4.4 mmol/L). In addition, the 3D model resulted in more aggressive insulin dosing and higher feed rates for similar intervention workload. Higher caloric intake is associated with improved outcomes [39–42]. These outcomes confirm the 3D stochastic model, using prior information in SI variability, and achieve effective control for all patients using more aggressive insulin dosing without compromising safety. Hence, STAR-3D offers a more patient-specific control, better accounting for either stable or very variable patients, potentially resulting in improved patient outcomes.

More importantly, the slightly lower median BG using STAR-3D (6.3 [5.7, 7.0] vs. 6.2 [5.6, 6.9]) was achieved with significantly higher time (61% vs. 56%, *p* < 0.01 using *χ*^2^ statistical test on proportions of measurements) in the 4.4–6.5 mmol/L band and in the 4.4–7.0 mmol/L band (75% vs. 72%, *p* < 0.01 using *χ*^2^ statistical test on proportions of measurements). While the low values for these *p* values could be influenced by the large data set size [43, 44], this difference is also clinically significant, since larger values in these ranges have been associated with improved outcomes and higher odds of living [45–47]. In addition, there was a consistent, high, 88% BG in target band (4.4–8.0 mmol/L). High percentage time in these ranges has all been associated with improved clinical outcomes in multiple independent studies [21, 45–47]. These results, together with the minimal risk of hypoglycaemia (< 2%) and severe hyperglycaemia (< 3%), prove the STAR framework design to be adapted for GC in critical care, to provide safe, effective control for all patients, and show GC to lower target ranges to be possible without compromising safety.

It is also important to note specific safety benefits of this new model are hard to highlight. First of all, because hypoglycemia, in STAR, is extremely infrequent, unlike many other protocols failing to achieve safe control [12, 15, 48–51]. Hence, the few hours where the 3D model enables a gain in potentially very harmful hypoglycemia due to highly variable SI is hard to see and overwhelmed in the overall high effectiveness of STAR. Thus, we examine improved performance, which is beneficial to patients, with equivalent safety.

**Fig. 5:**
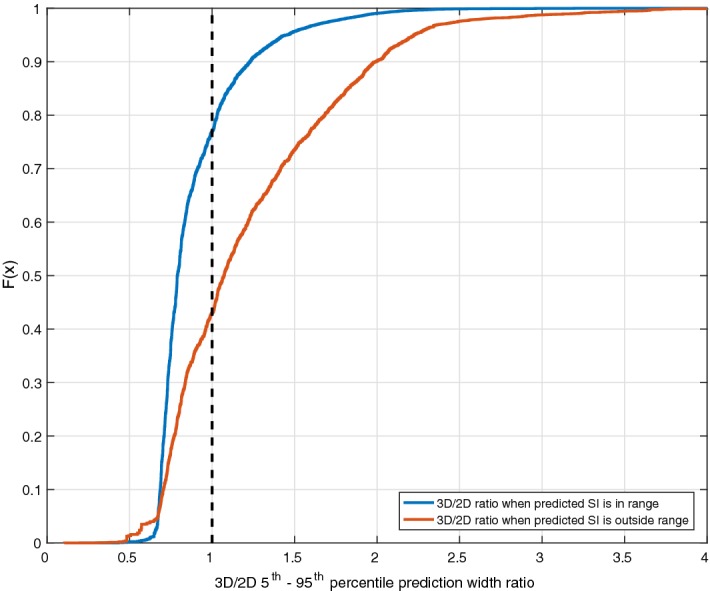
Prediction range ratios cumulative distribution functions when predicted SI is within predicted range (blue) or outside (red)

To further show this, the following cumulative distribution functions of the ratio between the 5th–95th percentile range widths of each model when the subsequent SI value is within the predicted range and when the prediction is outside this range is shown below (Fig. 5). When SI is within the predicted range (~ 90% of hours, Table 1), the 3D model prediction band is tighter > 75% of the time. However, when the subsequent SI value is outside the predicted range (~ 10% of the time), the 3D model is already > 55% of the time wider than the 2D model. This result suggests that when the subsequent SI value is outside the range, the 3D model is generally more conservative (with a wider interval predicted) despite SI being outside predicted range. However, when the subsequent SI is within the predicted range, it is far narrower. Thus, the 3D model is overall safer.

While the difference in the two models shown in Table 1 is quite important, and the virtual trials showed higher performance (Table 2), a greater difference in glycaemic outcomes might have been expected. First, this difference shows how the STAR framework is consistent and manages to control patients in a safe manner. Second, the difference in SI prediction ranges between these models may not be big enough to change the discretized insulin interventions in STAR, as the controller is limited to 0.5 U/h increments. More specifically, in [25], an analysis suggests that a change below 12–15% in SI levels can be considered clinically equivalent, limiting some impact on GC recommendations.

STAR treatment selection relies in putting the 5th percentile of predicted BG outcome on the lower target band limit. Hence, it mainly uses the 95th percentile of predicted SI. Looking deeper at the 95th percentile difference between the 2D and 3D model, there is median reduction of ~ 6%, which may not be enough to significantly change the administration rate of insulin.

As reflected in these results, using more information to better predict how likely patient-specific metabolic conditions will change seems a good approach to improve control in the STAR framework. More specifically, using prior state of identified SI values also allows to suffer less from direct measurement errors or identification errors for the future prediction [52]. While one could think to extend this method to more dimensions, the danger would be to over fit the data and/or suffer from low data density. Therefore, it could result in non-desired behaviour for higher computational costs. However, other parameters could be useful to improve both predictions and GC outcomes. In [53], BG data are used as an entry with current SI level to forecast metabolic variability. In doing so, not only it potentially can improve control safety and efficacy, but it also allows to identify specific behaviour in the data, reflected by the resulting estimated distributions. In particular, [53] observed typical underestimation of SI changes at lower BG values and vice versa. Hence, more work could be done to identify possible critical factors or parameters allowing to further improve prediction of important changes in metabolic variability and SI.

The bi-variate kernel-density estimation method requires much fewer total data to create an effective model for use in clinical practice compared to the tri-variate model presented. However, the 3D stochastic model demonstrated better performance and equivalent safety in this study due to the much higher number data triplets (~ 60,000 vs. ~ 20,000) available from the larger population data set used in this study than in creating the 2D stochastic model [34]. In addition, the equivalence across the virtual trial fivefold cross -validation results suggests that the stochastic models were created on enough data to be robust and that the data used were representative of a general ICU population.

The interpretation of these results has some limitations. Virtual trials represent realistic glycaemic outcomes in perfect conditions, fully compliant to the protocol [54]. Glycaemic outcomes will likely differ at least somewhat in a real clinical environment. However, these virtual trials have been validated and shown to well capture the overall potential glycaemic outcomes [55, 56]. In addition, compliance to STAR is very high in regular clinical use [28, 57].

## Conclusions

Tri-variate kernel-density estimation methods are used to build a new 3D stochastic model forecasting likely future changes in insulin sensitivity based on its prior 2 states. This 3D stochastic model shows similar, high, forward predictive power compared to the previous 2D version, but achieved with 15–25% tighter prediction ranges more than 70% of the time. This suggests that the 3D stochastic model better predicts future SI dynamics and thus offers greater personalisation of care than the prior 2D model.

Virtual trials using this model showed similar glycaemic control safety and better performance based on higher time in the normoglycaemic intermediate ranges (4.4–6.5 mmol/L and 4.4–7.0 mmol/L), resulting in slightly lower median BG levels for similar workload. These improvements are due to greater personalisation of care, and were achieved using higher insulin rates and slightly higher nutrition rates in cases where possible and as enabled by the tighter prediction ranges offered in over 70% of interventions. These results suggest that the implementation of this new 3D stochastic model within the STAR framework could potentially improve patient clinical outcomes resulting from improved glycaemic control.

## Methods

### Model-based insulin sensitivity

**Fig. 6:**
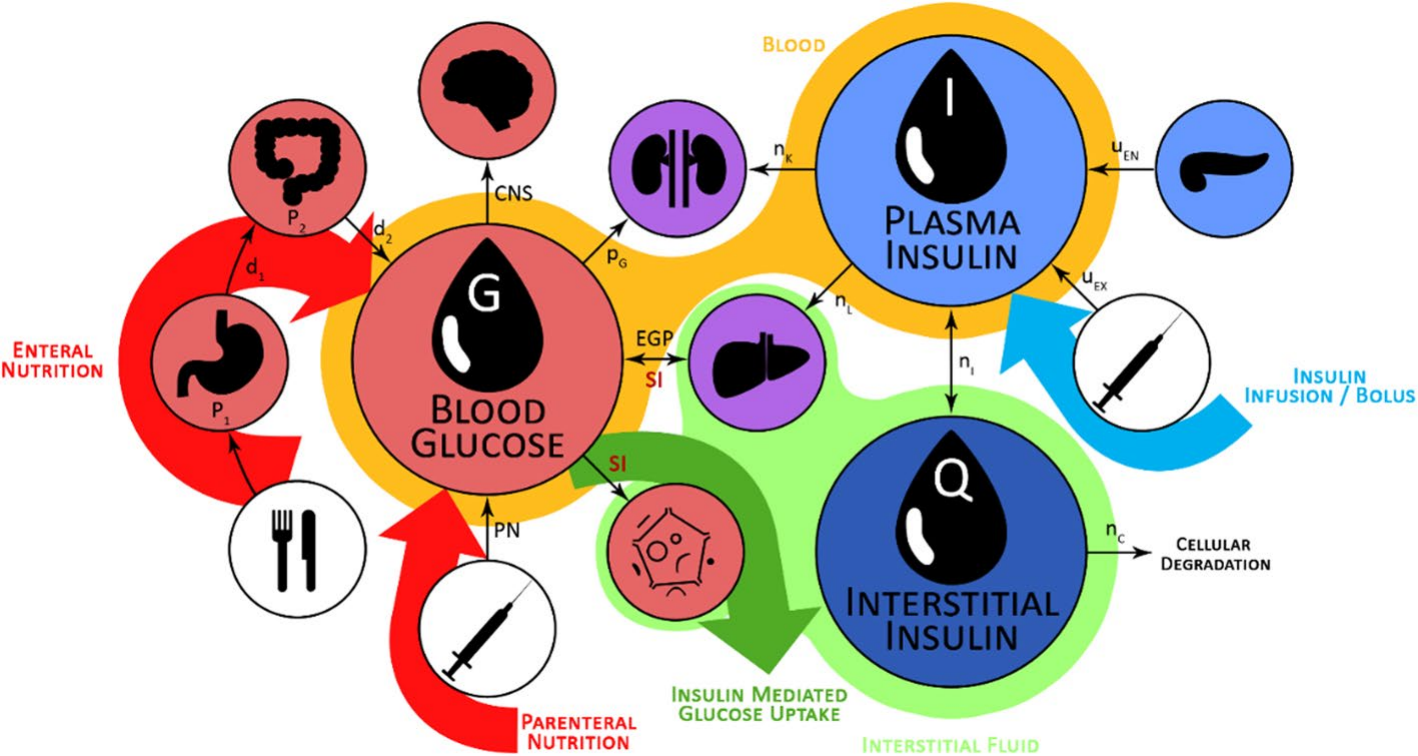
Schematic representation of the ICING model. Enteral and parenteral nutrition pathways are shown, as the endogenous and exogenous insulin contributions

The validated Intensive Control Insulin–Nutrition–Glucose physiological (ICING) [58] model used in STAR describes the glucose–insulin pharmacokinetics and can be graphically represented as the 3 compartment model in Fig. 6. It is defined:

1 $$\dot{G} = - p_{G} .G\left( t \right) - {\text{SI}}.G\left( t \right)\frac{Q\left( t \right)}{{1 + \alpha_{G} .Q\left( t \right)}} + \frac{{P\left( t \right) + {\text{EGP}} - {\text{CNS}}}}{{V_{G} }}$$

2 $$\dot{I} = - n_{K} .I\left( t \right) - n_{L} \frac{I\left( t \right)}{{1 + \alpha_{I} .I\left( t \right)}} - n_{I} \left( {I\left( t \right) - Q\left( t \right)} \right) + \frac{{u_{ex} \left( t \right)}}{{V_{I} }} + \left( {1 - x_{L} } \right)\frac{{u_{en} \left( G \right)}}{{V_{I} }}$$

3 $$\dot{Q} = n_{I} \left( {I\left( t \right) - Q\left( t \right)} \right) - n_{C} \frac{Q\left( t \right)}{{1 + \alpha_{G} Q\left( t \right)}},$$

where *G(t)* is the blood glucose concentration (mmol/L), *I(t)* and *Q(t)* are the plasma and interstitial insulin concentrations (mU/L), *P(t)* is the glucose appearance in plasma from enteral and parenteral dextrose intakes (mmol/min), and *SI* is the insulin sensitivity (L/mU/min). Other parameters are listed in Table 3. Clearance rates and constants can be found elsewhere [25, 52, 58].

**Table 3:** Parameters of the Intensive Control Insulin–Nutrition–Glucose physiological model (Eqs. 1–3)

$p_{G}$	Non-insulin-mediated glucose clearance
$\alpha_{G}$	Saturation of insulin-mediated glucose uptake
$EGP$	Endogenous Glucose Production (hepatic)
$CNS$	Glucose uptake by Central Nervous System
$V_{G}$	Glucose distribution volume
$n_{K}$	Kidney clearance of insulin
$n_{L}$	Liver clearance of insulin
$\alpha_{I}$	Saturation of hepatic insulin clearance
$n_{I}$	Insulin diffusion between plasma and interstitium
$n_{C}$	Cellular degradation of internalised insulin
$x_{L}$	Fractional first pass hepatic insulin clearance from portal vein
$V_{I}$	Insulin distribution volume
$u_{ex} \left( t \right)$	Exogenous insulin
$u_{en} \left( G \right)$	Endogenous insulin

The model-based time-varying SI parameter describes the patient-specific glycaemic metabolic response to insulin administration. SI is identified hourly from clinical BG, insulin, and nutritional data using integral-based fitting methods [59]. The validity of this SI metric is demonstrated in [25] for ICU patients, as well as in several clinical studies [60–62].

### Stochastic modelling

To account for patient-specific metabolic variability, and thus assess unexpected potential changes in metabolic response to insulin, [34] introduced a probabilistic model predicting likely future 1–3 hourly change in SI level (SI_*n*+1_, SI_*n*+2_, SI_*n*+3_). These predictions are only based on current identified patient metabolic condition (SI_*n*_). This stochastic model was built using a two-dimensional kernel-density estimation method on population data, and led to the emergence of the first successful risk-based dosing approach for GC [31, 63]. The kernel-density estimation method enables high-resolution behaviour estimation of a specific parameter based upon its prior evolution or state, even where specific data points may be scarce.

**Fig. 7:**
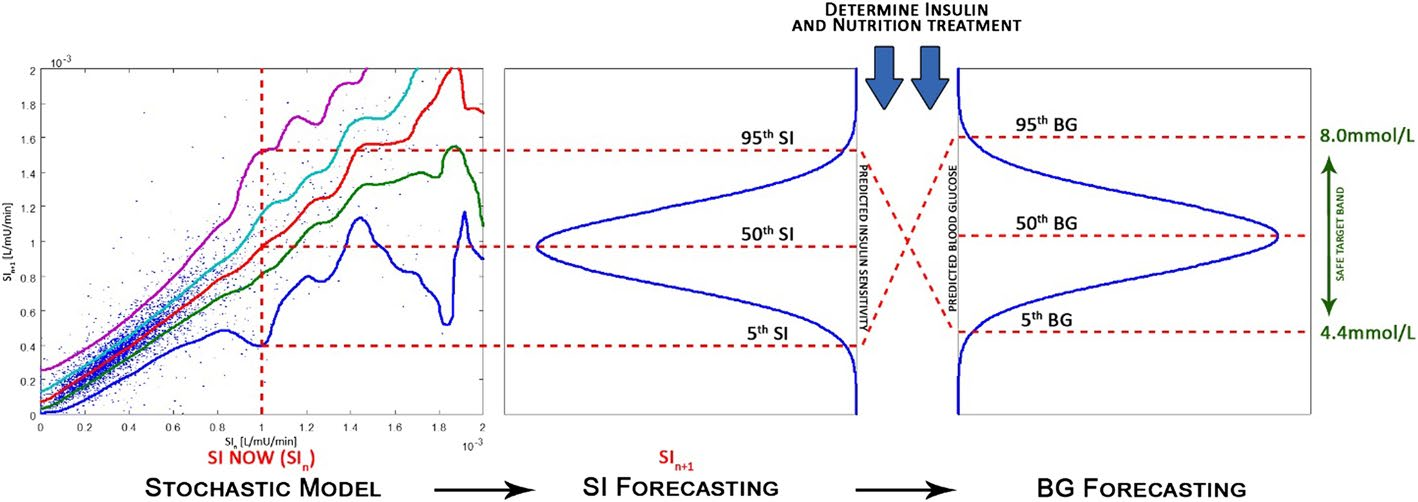
Risk-based dosing approach of the STAR framework. Current patient-specific identified SI is used to forecast the likely 5th–95th percentile range of future SI. This range is used to calculate the corresponding 5th–95th percentile range of likely future BG outcome for given insulin and nutrition inputs

Using the identified SI_*n*_, the 2D stochastic model forecasts likely future distribution of SI_*n*+1_, as graphically represented in Fig. 7. This likely future SI distribution allows prediction of the corresponding likely future BG distribution for a given insulin and nutrition intervention using Eqs. 1–3 (Fig. 7). Specifically, the 5th–95th percentile range of likely future SI is used to compute the corresponding 5th–95th percentile range of predicted future BG outcomes. STAR then adjusts treatments by ensuring the 5th and 95th percentiles of future BG lie within the clinically specified target range (4.4–8.0 mmol/L in STAR), minimizing the risk of BG < 4.4 mmol/L to 5% [31].

This study extends the bi-variate kernel estimation method to tri-variate. The predictions of future SI_*n*+1_, SI_*n*+2_, and SI_*n*+3_ are thus determined using two inputs to potentially increase patient-specific variability forecasting, which could also result in better overall safety and performance for STAR GC decision making. In particular, this choice of data triplets (SI_*n*−1_, SI_*n*_) → SI_*n*+1,2,3_, add patient specificity to the SI_*n*_ → SI_*n*+1,2,3_ 2D model by making these distributions a function of prior states. This difference thus includes a greater part of the patient-specific evolution, and thus will further characterise patients, creating greater personalisation in the GC predictions based on thus enhanced stochastic model. It thus assumes that there will be measurable differences in the predicted SI_*n*+1,2,3_ distributions by this added data, compared to those from the 2D model. Importantly, the 3D approach significantly increases the data requirements for model generation, resulting in the use of a much larger data set size (~ 60,000 h) compared to the previous studies [34].

### From data density to conditional probability

SI in this study can be considered a second-order finite Markov chain, where the current state depends only on its two prior states. Therefore, the conditional probability distribution of the future SI_*n*+1_ is a function of SI_*n*_ and SI_*n*-1_ states which can be expressed:

4 $$P\left( {{\text{SI}}_{n + 1} |{\text{SI}}_{n} ,{\text{SI}}_{n - 1} , \ldots ,{\text{SI}}_{0} } \right) = P\left( {{\text{SI}}_{n + 1} |{\text{SI}}_{n} ,{\text{SI}}_{n - 1} } \right) = \frac{{P({\text{SI}}_{n + 1} ,{\text{SI}}_{n} ,{\text{SI}}_{n - 1} )}}{{P({\text{SI}}_{n} ,{\text{SI}}_{n - 1} )}},$$

where the right-hand expression is derived from the general product rule. Kernel-density methods are used to estimate the joint probability $P({\text{SI}}_{n + 1} ,{\text{SI}}_{n} ,{\text{SI}}_{n - 1} )$ and $P({\text{SI}}_{n} ,{\text{SI}}_{n - 1} )$ using tri and bi-variate Gaussian kernel-density estimator functions [64]. Therefore, the conditional probability of SI_*n*+1_ taking a specific value can be calculated using the identified SI_*n*_ and SI_*n*-1_ values, such that

5 $$\frac{{P\left( {{\text{SI}}_{n + 1} = z|{\text{SI}}_{n} = y,{\text{SI}}_{n - 1} = x} \right)}}{{P\left( {{\text{SI}}_{n} = y,{\text{SI}}_{n - 1} = x} \right)}} = \frac{{\frac{1}{N}\sum\nolimits_{i = 1}^{N} {\frac{{K_{{hx_{i} }} \left( {u_{{x_{i} }} } \right)}}{{p_{{x_{i} }} }}} \frac{{K_{{hy_{i} }} \left( {u_{{y_{i} }} } \right)}}{{p_{{y_{i} }} }}\frac{{K_{{hz_{i} }} \left( {u_{{z_{i} }} } \right)}}{{p_{{z_{i} }} }}}}{{\frac{1}{N}\sum\nolimits_{j = 1}^{N} {\frac{{K_{{hx_{j} }} (u_{{x_{j} }} )}}{{p_{{x_{j} }} }}} \frac{{K_{{hy_{j} }} (u_{{y_{j} }} )}}{{p_{{y_{j} }} }}}},$$

where $K_{h} \left( u \right)$ denotes the Gaussian kernel-density function $K_{h} \left( u \right) = \frac{1}{{\sqrt {2\pi } h}}e^{{ - \frac{1}{2}\left( {\frac{u}{h}} \right)^{2} }}$, centered on *u* with variance *h*, constructed using the available *N* data points [38, 65]. To optimize the approximation of data behaviour, the variance *h*, or scale factor, is determined using the general Silverman’s ROT [38, 64], weighted according to local data density:

6 $$h = 0.9686\sigma \left( {\frac{{mR^{3} N^{3/7} }}{Z}} \right)^{ - 1/7} ,$$

where *m* is the number of data point within a radius $N^{ - 1/7}$ after orthonormalisation of the data [34], and R is the radius from the origin encompassing Z*N data points (0 ≤ *Z* ≤ 1). This rule assumes that data have an underlying normal distribution [38]. Non-negativity is ensured by normalizing each Gaussian function to the positive defined domain, such that for each $({\text{SI}}_{n} = y,{\text{SI}}_{n - 1} = x)$ pair, there exists an estimated conditional probability function $P\left( {{\text{SI}}_{n + 1} = z | {\text{SI}}_{n} = y,{\text{SI}}_{n - 1} = x} \right)$, where $\smallint P\left( {{\text{SI}}_{n + 1} = z | {\text{SI}}_{n} = y,{\text{SI}}_{n - 1} = x} \right){\text{d}}z = 1$ is satisfied [64]. Normalization is achieved by dividing each kernel-density function $K_{hx_i} \left( u_{x_i} \right), K_{hy_i} \left( u_{y_i} \right), K_{hz_i} \left( u_{z_i} \right)$ in Eq. 5 by the area under each gaussian curve between zero and infinity:

7 $$p_{x_i} = \mathop \int \nolimits_{0}^{\infty } K_{hx_i} \left( u_{x_i} \right){\text{d}}x,\quad p_{y_i} = \mathop \int \nolimits_{0}^{\infty } K_{hy_i} \left( u_{y_i} \right){\text{d}}y,\quad {\text{and}} \quad p_{z_i} = \mathop \int \nolimits_{0}^{\infty } K_{hz_i} \left( u_{z_i} \right){\text{d}}z .$$

**Fig. 8:**
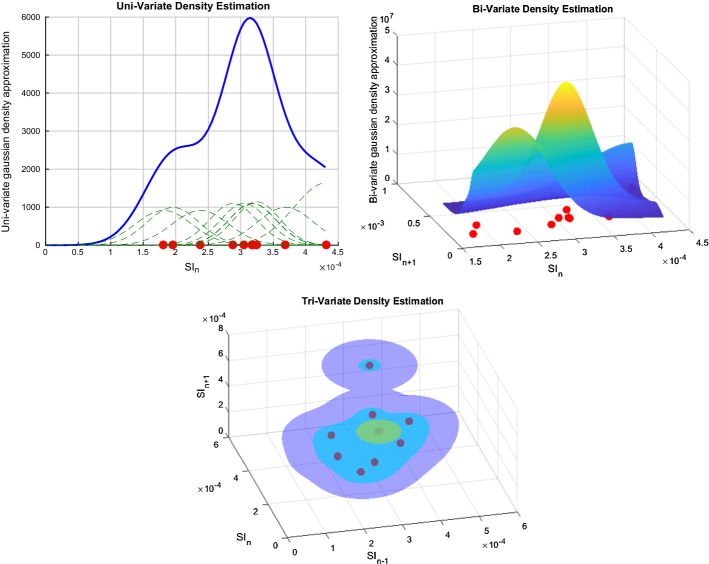
Uni-, bi-, and tri-variate kernel-density estimation for 10 data triplets. Dotted green lines show Gaussian distributions around each data point, where the standard deviation is a function of data density

This forces *x*, *y*, and *z* to be ≥ 0, ensuring thus physiological validity of SI values ≥ 0. An example of the resulting uni-, bi-, and tri-variate Gaussian kernel-density estimation for 10 data triplets is shown in Fig. 8.

### Patients and cohorts

This study uses clinical data from 606 patients across 3 different clinical trials and ICU settings (STAR Christchurch New Zealand 2011–2015, SPRINT Christchurch New Zealand 2005–2007, and STAR Gyula Hungary 2011–2015) [9, 28]. These data include 819 GC episodes and a total of 68,629 h of treatment. A patient can have multiple GC episodes, generally because:

1. Patients’ glycaemia is stabilized, but then several hours later GC is started again due to dysglycaemia arising from any potential clinical reason, or
2. Patients are sent out of the ICU for clinical procedures (most commonly imaging or surgery), where GC is stopped and started again as they return (if necessary).

Overall cohort demographics are shown in Table 4.

**Table 4:** Summary of patient demographic data. Data are given as median [IQR] where relevant

	SPRINT Christchurch	STAR Christchurch	STAR Gyula
# episodes	442	330	47
# patients	292	267	47
# hours	39,838	22,523	6268
% male	62.7	65.5	61.7
Age (years)	63 [48, 73]	65 [55, 72]	66 [58, 71]
APACHE II	19.0 [15.0, 24.5]	21.0 [16.0, 25.0]	32.0 [28.0, 36.0]
LOS–ICU (days)	6.2 [2.7, 13.0]	5.7 [2.5, 13.4]	14.0 [8.0, 20.5]

**Fig. 9:**
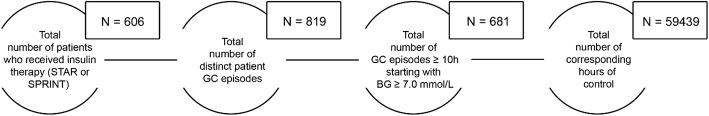
GC episode selection from the original 606 patients (819 different GC episodes)

From the original 819 episodes, only 681 episodes ≥ 10 h and with initial BG ≥ 7 mmol/L are considered (Fig. 9), corresponding to 59,439 h of control. These criteria ensure the exclusion of patient data with very short GC episodes, and thus low BG measurement numbers, or uncommonly low starting BG values likely less reflective of general metabolism dynamics. SI is identified hourly for each patient using integral-based fitting method and a total of 58,539, 57,840, and 57,141 data triplets (SI_*n*-1_, SI_*n*_, SI_*n*+*i*_) for *i* = 1, 2, and 3 h forward, respectively, are created.

### Validation and comparison analysis

The 2D and 3D stochastic models are built and compared using fivefold cross validation, where the resulting training (80%) and testing (20%) sets are believed to be statistically representative of the general data set, minimizing bias and variance in the validation [66]. Patients are thus randomly divided into 5 equally sized groups, models are built using 80% of patient episodes (4/5 groups), and the other 20% of patients (1/5 groups) are used for validation. As the Silverman’s ROT for multivariate kernel-density estimation assumes data has a Gaussian distribution [38], and SI has a log-normal distribution, the logarithmic domain is chosen here to build the model.

The 25th–75th and 5th–95th percentile ranges are computed for both models. Tighter prediction ranges for future SI_*n*+*i*_ would suggest likely lower future variability. In this case, the future potential variation for a given insulin dose being smaller, STAR can provide insulin with greater certainty, and thus potentially more aggressively (higher insulin rates) with equal safety. On the other hand, wider prediction bands would suggest higher future variability and, thus, more conservative dosing of insulin use to avoid hypoglycaemia. Forward predictive power and model accuracy are compared using the percentage of accurate predictions within these two ranges. The expected accuracies are 50% and 90%, respectively, where conformation of an independent cohort to these expected outcomes would indicate the 3D methods accurately capture SI dynamics to predict future SI.

Finally, to assess clinical impact, validated virtual trials on virtual patients are simulated to assess the new models ability to control patients. Such virtual trials enable comparison of glycaemic outcomes from different GC designs, on the same underlying patients. In summary, virtual patients are characterised by their identified patient-specific SI traces generated from clinical data, and can be used to test a range of new protocols or technologies [67, 68]. They are well-validated in their independence from the data used to create them and their accuracy [56, 69], their ability to predict trial outcomes [63, 70] and in clinical use to guide care in STAR [28, 31]. The underlying model is also well-validated in insulin sensitivity testing and similar clinical studies [60, 71, 72]. These virtual trials have been validated in the previous studies [55, 56], and are used here to simulate STAR using either the 2D (STAR-2D) or 3D (STAR-3D) stochastic model.

Unlike most GC protocols, STAR has the ability to modulate both insulin and nutrition inputs. Enteral nutrition can be lowered if the maximum allowed insulin is not sufficient to decrease BG levels, often occurring for very resistant patients with low SI and saturation of insulin-dosing effects. In STAR, insulin is administered as boluses up to a maximum of 6 U/h, with an additional 3 U/h continuous infusion for highly resistant patients. Enteral nutrition administration can be modulated between 30 and 100% of the total calorific goal feed (GF) if necessary. The original 100% GF for a patient is computed according to the standard 25 kcal/kg/day target adapted based on age and sex. Further details are in [28, 42].

Safety and performance, administered insulin, and nutrition delivery are compared from these simulations. BG is resampled hourly, to allow fair comparison across the different measurement intervals. Safety is assessed by the %BG in mild (%BG ≤ 4.4 mmol/L) and severe (%BG ≤ 2.2 mmol/L) hypoglycaemia and in hyperglycaemia (%BG > 8.0 mmol/L and %BG > 10.0 mmol/L). Performance is assessed by the %BG in target band (4.4–8.0 mmol/L) and the median [IQR] BG levels achieved. Nutrition is reported as the percentage goal feed (%GF) achieved. In addition, workload is also compared as the number of BG measurement per day, where a higher value indicates increased workload.

## Data Availability

The data sets used and/or analysed during the current study are available from the corresponding author on reasonable request. However, a subset of the data is publicly available in another journal: Chase J.G. et al. A benchmark data set for model-based glycemic control in critical care, J Diabetes Sci Technol 2008, 2(4): 584–94.

## Abbreviations

BG: blood glucose
GC: glycaemic control
ICING: Intensive Control Insulin–Nutrition–Glucose
ICU: intensive care unit
IQR: interquartile range
ROT: rule of thumb
STAR: stochastic targeted
SI: insulin sensitivity

## References

[1] McCowen KC, Malhotra A, Bistrian BR (2001). Stress-induced hyperglycemia. Crit Care Clin.

[2] Ali NA, O’Brien JM, Dungan K, Phillips G, Marsh CB, Lemeshow S, Connors AF, Preiser JC (2008). Glucose variability and mortality in patients with sepsis. Crit Care Med.

[3] Krinsley JS (2009). Glycemic variability and mortality in critically ill patients: the impact of diabetes. J Diabetes Sci Technol.

[4] Capes SE, Hunt D, Malmberg K, Gerstein HC (2000). Stress hyperglycaemia and increased risk of death after myocardial infarction in patients with and without diabetes: a systematic overview. Lancet.

[5] Van den Berghe G, Wouters P, Weekers F, Verwaest C, Bruyninckx F, Schetz M, Vlasselaers D, Ferdinande P, Lauwers P, Bouillon R (2001). Intensive insulin therapy in critically ill patients. N Engl J Med.

[6] Krinsley JS (2004). Effect of an intensive glucose management protocol on the mortality of critically ill adult patients. Mayo Clin Proc.

[7] Reed CC, Stewart RM, Sherman M, Myers JG, Corneille MG, Larson N, Gerhardt S, Beadle R, Gamboa C, Dent D (2007). Intensive insulin protocol improves glucose control and is associated with a reduction in intensive care unit mortality. J Am Coll Surg.

[8] Chase JG, Pretty CG, Pfeifer L, Shaw GM, Preiser JC, Le Compte AJ, Lin J, Hewett D, Moorhead KT, Desaive T (2010). Organ failure and tight glycemic control in the SPRINT study. Crit Care.

[9] Chase JG, Shaw G, Le Compte A, Lonergan T, Willacy M, Wong XW, Lin J, Lotz T, Lee D, Hann C (2008). Implementation and evaluation of the SPRINT protocol for tight glycaemic control in critically ill patients: a clinical practice change. Crit Care.

[10] Krinsley JS (1995). Is glycemic control of the critically ill cost-effective?. Hosp Pract.

[11] Finfer S, Chittock D, Li Y, Foster D, Dhingra V, Bellomo R, Cook D, Dodek P, Hebert P, Henderson W (2015). Intensive versus conventional glucose control in critically ill patients with traumatic brain injury: long-term follow-up of a subgroup of patients from the NICE-SUGAR study. Intensive Care Med.

[12] Brunkhorst FM, Engel C, Bloos F, Meier-Hellmann A, Ragaller M, Weiler N, Moerer O, Gruendling M, Oppert M, Grond S (2008). Intensive insulin therapy and pentastarch resuscitation in severe sepsis. N Engl J Med.

[13] Signal M, Fisk L, Shaw GM, Chase JG (2013). Concurrent continuous glucose monitoring in critically Ill patients: interim results and observations. J Diabetes Sci Technol.

[14] Signal M, Pretty CG, Chase JG, Le Compte A, Shaw GM (2010). Continuous glucose monitors and the burden of tight glycemic control in critical care: can they cure the time cost?. J Diabetes Sci Technol.

[15] Preiser JC, Devos P, Ruiz-Santana S, Melot C, Annane D, Groeneveld J, Iapichino G, Leverve X, Nitenberg G, Singer P (2009). A prospective randomised multi-centre controlled trial on tight glucose control by intensive insulin therapy in adult intensive care units: the Glucontrol study. Intensive Care Med.

[16] Van Herpe T, De Moor B, Van den Berghe G (2009). Ingredients for adequate evaluation of blood glucose algorithms as applied to the critically ill. Crit Care.

[17] Bagshaw SM, Bellomo R, Jacka MJ, Egi M, Hart GK, George C, Committee ACM (2009). The impact of early hypoglycemia and blood glucose variability on outcome in critical illness. Crit Care.

[18] Egi M, Bellomo R, Stachowski E, French CJ, Hart G (2006). Variability of blood glucose concentration and short-term mortality in critically ill patients. Anesthesiology.

[19] Egi M, Bellomo R, Stachowski E, French CJ, Hart GK, Taori G, Hegarty C, Bailey M (2010). Hypoglycemia and outcome in critically ill patients. Mayo Clin Proc.

[20] Krinsley JS (2003). Association between hyperglycemia and increased hospital mortality in a heterogeneous population of critically ill patients. Mayo Clin Proc.

[21] Penning S, Pretty C, Preiser JC, Shaw GM, Desaive T, Chase JG (2015). Glucose control positively influences patient outcome: a retrospective study. J Crit Care.

[22] Chase JG, Le Compte AJ, Suhaimi F, Shaw GM, Lynn A, Lin J, Pretty CG, Razak N, Parente JD, Hann CE (2011). Tight glycemic control in critical care–the leading role of insulin sensitivity and patient variability: a review and model-based analysis. Comput Methods Programs Biomed.

[23] Krinsley JS (2018). Is it time to rethink blood glucose targets in critically ill patients?. Chest.

[24] Roubicek T, Kremen J, Blaha J, Matias M, Kopecky P, Rulisek J, Anderlova K, Bosanska L, Mraz M, Chassin LJ (2007). Pilot study to evaluate blood glucose control by a model predictive control algorithm with variable sampling rate vs. routine glucose management protocol in peri- and postoperative period in cardiac surgery patients. Cas Lek Cesk.

[25] Kuure-Kinsey M, Palerm CC, Bequette BW (2006). A dual-rate Kalman filter for continuous glucose monitoring. Conf Proc IEEE Eng Med Biol Soc.

[26] Reifman J, Rajaraman S, Gribok A, Ward WK (2007). Predictive monitoring for improved management of glucose levels. J Diabetes Sci Technol.

[27] Uyttendaele V, Knopp JL, Shaw GM, Desaive T, Chase JG (2019). Is intensive insulin therapy the scapegoat for or cause of hypoglycaemia and poor outcome?. IFAC J Syst Control.

[28] Stewart KW, Pretty CG, Tomlinson H, Thomas FL, Homlok J, Noemi SN, Illyes A, Shaw GM, Benyo B, Chase JG (2016). Safety, efficacy and clinical generalization of the STAR protocol: a retrospective analysis. Ann Intensive Care.

[29] Lunn DJ, Wei C, Hovorka R (2011). Fitting dynamic models with forcing functions: application to continuous glucose monitoring in insulin therapy. Stat Med.

[30] Schultz MJ, Harmsen RE, Korevaar JC, Abu-Hanna A, Van Braam Houckgeest F, Van Der Sluijs JP, Spronk PE (2012). Adoption and implementation of the original strict glycemic control guideline is feasible and safe in adult critically ill patients. Minerva Anestesiol.

[31] Evans A, Le Compte A, Tan CS, Ward L, Steel J, Pretty CG, Penning S, Suhaimi F, Shaw GM, Desaive T, Chase JG (2012). Stochastic targeted (STAR) glycemic control: design, safety, and performance. J Diabetes Sci Technol.

[32] Suhaimi F, Le Compte A, Preiser JC, Shaw GM, Massion P, Radermecker R, Pretty CG, Lin J, Desaive T, Chase JG (2010). What makes tight glycemic control tight? The impact of variability and nutrition in two clinical studies. J Diabetes Sci Technol.

[33] Uyttendaele V, Dickson JL, Shaw GM, Desaive T, Chase JG (2017). Untangling glycaemia and mortality in critical care. Crit Care.

[34] Lin J, Lee D, Chase JG, Shaw GM, Le Compte A, Lotz T, Wong J, Lonergan T, Hann CE (2008). Stochastic modelling of insulin sensitivity and adaptive glycemic control for critical care. Comput Methods Programs Biomed.

[35] Lin J, Lee D, Chase JG, Shaw GM, Hann CE, Lotz T, Wong J (2006). Stochastic modelling of insulin sensitivity variability in critical care. Biomed Signal Process Control.

[36] Vanhorebeek I, Langouche L, Van den Berghe G (2005). Glycemic and nonglycemic effects of insulin: how do they contribute to a better outcome of critical illness?. Curr Opin Crit Care.

[37] Uyttendaele V, Knopp JL, Stewart K, Desaive T, Benyo B, Szabo-Nemedy N, Illyes A, Shaw G, Chase JG (2018). A 3D insulin sensitivity prediction model enables more patient-specific prediction and model-based glycaemic control. Biomed Signal Process Control.

[38] Sheather SJ: Density estimation. In: *Statistical science. Volume* 19: Institute of mathematical statistics; 2004.[JSTOR (Series Editor).

[39] Villet S, Chiolero RL, Bollmann MD, Revelly JP, Cayeux RNM, Delarue J, Berger MM (2005). Negative impact of hypocaloric feeding and energy balance on clinical outcome in ICU patients. Clin Nutr.

[40] Krishnan JA, Parce PB, Martinez A, Diette GB, Brower RG (2003). Caloric intake in medical ICU patients: consistency of care with guidelines and relationship to clinical outcomes. Chest.

[41] Heyland DK, Cahill N, Day AG (2011). Optimal amount of calories for critically ill patients: depends on how you slice the cake!. Crit Care Med.

[42] Stewart KW, Chase JG, Pretty CG, Shaw GM (2018). Nutrition delivery of a model-based ICU glycaemic control system. Ann Intensive Care.

[43] Goodman SN (1999). Toward evidence-based medical statistics. 1: The P value fallacy. Ann Intern Med.

[44] Motulsky H (2015). Common misconceptions about data analysis and statistics. Br J Pharmacol.

[45] Signal M, Le Compte A, Shaw GM, Chase JG (2012). Glycemic levels in critically ill patients: are normoglycemia and low variability associated with improved outcomes?. J Diabetes Sci Technol.

[46] Penning S, Chase JG, Preiser JC, Pretty CG, Signal M, Melot C, Desaive T (2014). Does the achievement of an intermediate glycemic target reduce organ failure and mortality? A post hoc analysis of the Glucontrol trial. J Crit Care.

[47] Krinsley JS, Preiser JC (2015). Time in blood glucose range 70 to 140 mg/dl > 80% is strongly associated with increased survival in non-diabetic critically ill adults. Crit Care.

[48] Finfer S, Chittock DR, Su SY, Blair D, Foster D, Dhingra V, Bellomo R, Cook D, Dodek P, Henderson WR (2009). Intensive versus conventional glucose control in critically ill patients. N Engl J Med.

[49] Arabi YM, Dabbagh OC, Tamim HM, Al-Shimemeri AA, Memish ZA, Haddad SH (2008). Intensive versus conventional insulin therapy: a randomized controlled trial in medical and surgical critically ill patients. Crit Care Med.

[50] Rosa C, Donado JH, Restrepo AH, Quintero AM, Gonzalez LG, Saldarriaga NE (2008). Strict glycaemic control in patients hospitalised in a mixed medical and surgical intensive care unit: a randomised clinical trial. Crit Care.

[51] Treggiari MM, Karir V, Yanez ND, Weiss NS, Daniel S, Deem SA (2008). Intensive insulin therapy and mortality in critically ill patients. Crit Care.

[52] Pretty CG, Signal M, Fisk L, Penning S, Le Compte A, Shaw GM, Desaive T, Chase JG (2014). Impact of sensor and measurement timing errors on model-based insulin sensitivity. Comput Methods Programs Biomed.

[53] Marik PE, Preiser JC (2010). Toward understanding tight glycemic control in the ICU: a systematic review and metaanalysis. Chest.

[54] Chase JG, Andreassen S, Jensen K, Shaw GM (2008). Impact of human factors on clinical protocol performance: a proposed assessment framework and case examples. J Diabetes Sci Technol.

[55] Dickson JL, Stewart KW, Pretty CG, Flechet M, Desaive T, Penning S, Lambermont BC, Benyo B, Shaw GM, Chase JG (2018). Generalisability of a virtual trials method for glycaemic control in intensive care. IEEE Trans Biomed Eng.

[56] Chase JG, Suhaimi F, Penning S, Preiser JC, Le Compte AJ, Lin J, Pretty CG, Shaw GM, Moorhead KT, Desaive T (2010). Validation of a model-based virtual trials method for tight glycemic control in intensive care. Biomed Eng Online.

[57] Dickson J, Chase JG (2019). Clinical Compliance in Personalised Model-based Medical Decision Support: do computers and interfaces yield better compliance?. IFAC-PapersOnLine.

[58] Lin J, Razak NN, Pretty CG, Le Compte A, Docherty P, Parente JD, Shaw GM, Hann CE, Geoffrey Chase J (2011). A physiological Intensive Control Insulin–Nutrition–Glucose (ICING) model validated in critically ill patients. Comput Methods Programs Biomed.

[59] Dandona P, Mohanty P, Chaudhuri A, Garg R, Aljada A (2005). Insulin infusion in acute illness. J Clin Invest.

[60] McAuley KA, Berkeley JE, Docherty PD, Lotz TF, Te Morenga LA, Shaw GM, Williams SM, Chase JG, Mann JI (2011). The dynamic insulin sensitivity and secretion test—a novel measure of insulin sensitivity. Metabolism.

[61] Docherty PD, Chase JG, Lotz T, Hann CE, Shaw GM, Berkeley JE, Mann JI, McAuley K (2009). DISTq: an iterative analysis of glucose data for low-cost, real-time and accurate estimation of insulin sensitivity. Open Med Inform J.

[62] Docherty PD, Chase JG, Te Morenga L, Fisk LM (2014). A novel hierarchal-based approach to measure insulin sensitivity and secretion in at-risk populations. J Diabetes Sci Technol.

[63] Fisk LM, Le Compte AJ, Shaw GM, Penning S, Desaive T, Chase JG (2012). STAR development and protocol comparison. IEEE Trans Biomed Eng.

[64] Silverman BW (1986). Density estimation for statistics and data analysis.

[65] Scott DW, Gentle JE, Härdle WK, Mori Y (2012). Multivariate density estimation and visualization. Handbook of computational statistics: concepts and methods.

[66] James G, Witten D, Hastie T, Tibshirani R (2013). Resampling methods. An introduction to statistical learning.

[67] Chase JG, Preiser JC, Dickson JL, Pironet A, Chiew YS, Pretty CG, Shaw GM, Benyo B, Moeller K, Safaei S (2018). Next-generation, personalised, model-based critical care medicine: a state-of-the art review of in silico virtual patient models, methods, and cohorts, and how to validation them. Biomed Eng Online.

[68] Chase JG, Benyo B, Desaive T: Glycemic control in the intensive care unit: a control systems perspective. *Annual Reviews in Control* 2019.

[69] Dickson JL, Stewart KW, Pretty CG, Flechet M, Desaive T, Penning S, Lambermont BC, Benyo B, Shaw GM, Chase G. Generalisability of a virtual trials method for glycaemic control in intensive care. In: IEEE transactions on biomedical engineering 2017, p. 1. 10.1109/TBME.2017.2686432 28358672

[70] Lonergan T, Le Compte A, Willacy M, Chase JG, Shaw GM, Wong XW, Lotz T, Lin J, Hann CE (2006). A simple insulin-nutrition protocol for tight glycemic control in critical illness: development and protocol comparison. Diabetes Technol Ther.

[71] Docherty PD, Chase JG, David T (2012). Characterisation of the iterative integral parameter identification method. Med Biol Eng Comput.

[72] Docherty PD, Chase JG, Lotz TF, Hann CE, Shaw GM, Berkeley JE (2011). Independent cohort cross-validation of the real-time DISTq estimation of insulin sensitivity. Comput Methods Programs Biomed.

